# Hospital and Institutional News

**Published:** 1913-12-13

**Authors:** 


### HOSPITAL AND INSTITUTIONAL NEWS.

### "THE HOSPITAL'S" CHRISTMAS SUPPLEMENT.

Next week, as usual, The Hospital will publish a Special Appeal Number, in which will be given the results of the competition by prize essays which have been invited on "How to Spend Christmas in a Hospital," from the five points of view of the resident medical staff, the secretariat, matron, sister, or nurse. The last day for sending in such essays was December 11, and with regard to them it must be remembered that the Editor, of course, possesses the right not to award a prize if the essays sent in from any of the points of view are not up to the standard. In addition to the prize essays and the awards, however, which are a new feature, the Christmas Appeal Number, which is published as a Supplement separately, at the price of one penny, acts as a guide to the needs of the London hospitals, and enables readers to choose between the classified names of those which seem to them most worthy of support. The wider this Appeal Number is distributed, the wider will the claims of the voluntary hospitals be recognised at this charity-giving season of Christmas.

### SIR HENRY BURDETT'S REPORTS.

The *East Anglian Daily Press*, in republishing from The Hospital the report on the Cromer Cottage Hospital, makes the following editorial comment: "It is evidently time that something were done at Norwich and Lowestoft. Cromer, anyhow, can congratulate itself upon passing the expert censor so well: and it is worth while noting, in these days when some people still believe that women are incapable of dealing with public affairs, that the Cromer Hospital, 'one of the best of existing Cottage Hospitals,' is managed by a committee of fifteen women." All honour to the women of Cromer. May their example and success stimulate a movement amongst the women of the British Isles in favour of the voluntary hospitals.

### THE BRITISH HOSPITALS ASSOCIATION MEETING, 1914.

The British Hospitals Association has received an invitation to hold its annual Conference at Newcastle-on-Tyne, on a date, presumably in June, to be fixed by the Lord Mayor. There is probably no place of greater interest than Newcastle at the present moment to the managers and officials of the voluntary hospitals. Mr. Roden Orde and successive matrons at Newcastle have by great self-denial and marked ability brought the hospital, which had many defects and shortcomings as originally planned, into nearly perfect order. There is much to learn of value in connection with the management of the laundry, and the organisation of the departments where trained artificers and handicraftsmen are continuously employed, whilst the thorough mastery of all that is most essential in the electric lighting, sanitation, and drainage installations and equipment acquired by Mr. Orde will afford a multiplicity of constructive and executive detail full of practical value to all engaged in the management of a voluntary hospital. We hope that National Insurance and its effects upon voluntary hospital administration and work will form the chief subject for debate and examination. The voluntary hospitals are not receiving their due, and, as the financial foundation on which National Insurance rests in this country to-day, the time is rapidly approaching when they will all have to combine in the interests of the patients and the prevention of their abuse by defective State machinery and injustice. We congratulate all concerned upon the selection of Newcastle for the next Conference of the British Hospitals Association, which should prove fruitful in results so far as the voluntary hospitals and the working-classes are concerned. It is of the first importance to the artisan classes throughout the British Islands that National Insurance should be made a strength, and not a source of weakness to the British hospital system, upon which every industrial worker and his family is dependent in the day of serious illness or accident.

### THE LONDON HOSPITAL.

The Hon. Sydney Holland and the London Hospital are to be congratulated upon having raised £70,000 this year for their quinquennial appeal. Thirty thousand pounds more is required to complete the £100,000 essential to enable the committee of the London to meet its liabilities during the next five years. This £30,000 should be contributed without hesitation by the benevolent before the end of 1913, seeing that, despite the high state of efficiency which is maintained throughout the London Hospital, the sum asked for this quinquennium is £25,000 less than on former occasions. This fact points to energetic administration and the enforcement of business control which should appeal to every man of business and every woman who has learnt the importance and comfort of economy as opposed to parsimony in household affairs.

### A POSER FOR THE DEANS.

Our letter-box brings us many sidelights on the infinite variety of requirements which the human animal evolves in the course of his progress on this planet. One correspondent writes (the italics are his): "Having regard to the shortage of doctors, and having studied *for a hobby* for many years past medical and physiological treatises, I take the opportunity of inquiring whether you think there would be vacant at an early date a suitable salaried position in connection with any of the medical institutions, etc., where facilities for proceeding with medical studies would also be obtainable?" We fear the Deans will not all rush at this correspondent and fulfil all his desires. However, we shall, of course, be glad to forward any letters we may receive on the subject to the gentleman in question.

### INFIRMARY CHAIRMAN ASSAULTED.

A case which has excited more than local interest was heard at the Acton Police Court last week, when Dr. W. H. Williams prosecuted the husband of a female patient for causing him grievous bodily harm. The case centred around the doctor's attendance at the confinement of the defendant's wife, when the defendant alleged that his wife was insulted. Getting the doctor to come out of his house by a subterfuge, the defendant assaulted him with a walking-stick. Dr. Williams promptly prosecuted, and as the result of a full hearing at the Acton Police Court, the defendant withdrew all suggestions against the doctor, and fully apologised. The Magistrates fined the defendant £5, informed him he was lucky that Dr. Williams was not a vindictive man, and added that Dr. Williams left the Court without a stain on his character. Dr. Williams, who is a prominent figure in Acton, is a member of the Brentford Board of Guardians and chairman of the infirmary committee. He is also one of the hon. medical staff of the Acton Cottage Hospital, and has naturally received many expressions of sympathy and congratulation on his successful issue from this disgraceful attempt on his person and character.

### VENEREAL DISEASES: AN AUSTRALIAN EXPERIMENT.

The difficulties surrounding any public concerted measures to deal with syphilis in a country where no general steps have been taken hitherto lends additional interest to the description by Dr. Burnett Ham, at the sixth meeting of the Royal Commission on Venereal Diseases, of the measures recently adopted in Victoria. He said that the first step was to make syphilis notifiable in Melbourne metropolitan area, though no penalties enforced it and no names were given. In the course of a year 5,500 notifications were made, three-fifths of which were by private practitioners and the remaining 2,000 by the hospitals. As a result of tests it was calculated that the number of cases was equivalent to 0.5 per cent. of the population, a much lower figure than was expected. By the advice of the Advisory Committee the Government provided twenty-four free beds for men at the Alfred Hospital and twenty for women at the Women's Hospital, together with up-to-date equipment. When it became known that the treatment was in a general ward, and that this class of patient would not be dealt with differently from patients in the other wards, the first difficulty in filling them gave way to a steady demand. Another means of popularising the arrangements was the establishment of a night clinic at the Alfred Hospital. The general effect of these experimental arrangements was in Dr. Ham's view a greater general interest in the subject, with the result of securing early diagnosis and treatment. The importance of these results practically needs no emphasis, but indirectly they have probably paved the way for a Bill now before the Victorian Legislature, which embodies proposals for adequate free treatment, for prohibiting unqualified treatment, and for making the transmission of the disease knowingly a penal offence. These proposals will sound less startling to us when once the first prejudice has been removed by a public attempt at experimental measures.

### THE FATE OF THE AMBULANCE SCHEME.

We are sorry to learn that, after all, the question of the London Ambulance Service is not yet settled, the scheme of the Metropolitan Asylums Board, of which a full summary was published in last week's issue of The Hospital, having been vetoed by the General Purposes Committee of the London County Council, which is practically the Cabinet of that body. At Tuesday's meeting of the Council, Major Ernest Gray, chairman of the General Purposes Committee, announced that the Metropolitan Asylums Board's scheme had been submitted to the Committee, but they regretted that they could not recommend the Council to adopt it. A sub-committee had been appointed, and he hoped that it would be in a position to submit an alternative scheme to the Council at its next meeting. We presume that the financial outlay which would be involved is the stumbling-block, and it will be interesting to see how the Council itself will now tackle the matter. The first result is that further delay is inevitable.

### A COTTAGE HOSPITAL CLOSED.

A curious state of affairs has arisen in connection with Wellington Cottage Hospital, which, apparently, was erected over nine months ago, and is now to be closed on the ground of lack of public support. The provisional committee, which has come to this conclusion, is criticised by the Rev. Henry Cooper, who questions their power to act, and contends that the public has not had adequate opportunity for expressing its opinions. From what he said, it would seem that the public meeting held last month was not sufficiently representative, and that it was, in fact, a deadlock through the absence of those most qualified to discuss and guarantee adequate public support. The day has gone by when cottage hospital schemes can be taken up lightly and abandoned, and most people will agree with Mr. Cooper that it is "lamentable that the bequest of £3,400 for a cottage hospital for Wellington should mean only a closed building." What is the explanation of this extraordinary impasse?

### SCHOOL MEDICAL OFFICERS AT LOGGERHEADS.

Friction has become so acute between the school medical officer and the assistant school medical officer to the Cheltenham Education Committee that it has even reached Sir George Newman, chief medical officer to the Board of Education, and the deputation from the medical sub-committee which interviewed him has now presented its report. It appears that Dr. Garrett, the school medical officer, has resigned because the committee has not discharged Dr. Alice Burn, his assistant, for alleged insubordination. Both had been separately received by Sir George, who pointed out to the sub-committee that it was primarily for them to settle, and did not strictly enter his province until he had to advise the Board as to the issue of education grants, over which, he added, there might be serious difficulty if the present state of affairs continued. He offered, if desired, to give assistance and advice, and gave his opinion that an assistant school officer could not be allowed publicly to advocate personal views on food reform and such matters, nor to engage in controversial political movements, without the consent of those in authority. Dr. Garrett was therefore fully entitled to require Dr. Burn to refrain from doing so. That the latter's conduct amounted to technical insubordination he agreed, and thought that she should apologise to Dr. Garrett and undertake to work loyally with him in future. In the correspondence, unnecessarily voluminous, between the two officers, he thought there had been provocation on both sides. At the meeting to which this report was presented Dr. Blakeney moved that Dr. Burn apologise or else resign, and this was eventually carried. The great local interest excited over this storm in a tea-cup no doubt was encouraged by the sex question, which was unhappily introduced. Institutional life depends upon the successful working together of the two sexes, and it is a great pity that the co-operation which is so general in hospital work should break down in an allied branch where men and women can both bring their special aptitudes to bear also. The point to keep clear in this case is that it is a difference between individuals and not sexes which produced the trouble.

### THE SIGHT OF THE BLIND.

Nothing is more curious than the way, well known to scientists, in which one sense will, as it were, take the place of another, and adapt its powers under pressure of need. An interesting example of this is seen in the clay models of animals and other objects in the Sunderland Public Libraries, Museum, and Art Gallery, which have been made without assistance by the children of the County Blind School. The models are the outcome of a suggestion by Mr. J. A. Charlton Deas, librarian and director, who delivered a lecture in July on "How we May Show our Museums and Art Galleries to the Blind." During the King's and Queen's visit to Lord Durham the models were sent for them to see, and Lord Durham has written to Mr. Deas of their Majesties' interest in "these examples of the remarkable intelligence and skill displayed in the production of such accurate and life-like models of objects whose reality the pupils could only gauge by the delicacy of their touch." It appears also that Mr. Deas' system of instruction is being adopted in other museums on behalf of the blind. The interchange of the senses is a fascinating subject, and we remember having watched with interest an artist who used, when listening to music, to convey his sound-impressions into drawings, sometimes extraordinarily suggestive of the music we had heard in his company. How little paralysing the absence, if not the loss, of one sense may be all readers of Mr. H. G. Wells' "Country of the Blind" will remember.

### TELEPHONES AT NURSING HOMES.

According to a correspondent in the *Times* of Tuesday last, no defect in the telephone, however slight, can be remedied if it occurs between Saturday evening and Monday morning. The Gerrard Exchange informed him that not a single person was available for putting the matter right. The matter was urgent, as the breakdown occurred in a nursing home, which was thereby isolated for a week-end from medical men, relatives and friends, to the possible danger of the patients and the certain anxiety and trouble of everyone else. The telephone has now become as much a necessary of civilised life as the railway and the telegraph, and it is essential that reasonable means to repair defects should be provided over Sunday.

### DEPARTMENTAL CHANGES AT THE LONDON.

We were compelled to hold over from our account of developments at the London Hospital last week the proposal to build a hostel for the lodgment of all the resident staff. The house doctors' bed and sitting rooms are scattered all over the hospital, and it is felt that this would be the only satisfactory solution of the difficulty. It would be satisfactory from the point of view of the resident doctors, and, moreover, it would set free space in the hospital which is much needed. To wait until it might be possible to build a hostel was felt inadvisable, and, accordingly, the work is proceeding of enlarging the departments mentioned, chiefly by giving certain of the house doctors a bed-sitting room instead of a bed and sitting room as heretofore. Another departmental change is foreshadowed through the effect of the pressure of work on the domestic side of the hospital. The laundry, which seven years ago was perfectly adequate to deal with the 45,000 pieces per week, is now incapable of dealing with the 60,000 pieces which are an average week's work at present. Arrangements are therefore being made for an increase, both of machinery and of space accommodation.

### THE END OF THE DIMOCK CASE.

The last chapter in the circumstances attending the death of Dr. Dimock has now, apparently, closed. The inquiry, which had been deferred to enable Dr. Willcox, the Home office analyst, to give the results of his examination of the body, was concluded at Stretham last week, and the jury returned a verdict of "Death from poison, self-administered while temporarily insane." In the course of his evidence, Dr. Willcox said he found 2.67 grains of morphine and 6.7 grains of veronal. One grain of salts of morphine might be a fatal dose to an adult who was not habituated to the drug. He thought it probable that Dr. Dimock took the dose of veronal the night before he died, and the morphine, which was much quicker in action, within an hour of the time when his sister called him at 9 o'clock. After refusing permission to Mr. Copeman, who represented Dr. Meacock and other interested parties, to address the jury, the Coroner, Mr. G. M. Hall, said, in summing-up, that the charge of criminal libel had, no doubt, worried Dr. Dimock to excess. In justice to those who brought the charge, he added that a man did not set the criminal law in motion against a professional colleague without having strong evidence to support it. As the proceedings came to an end with his death, it was best for all parties to bury the trouble in the grave of the deceased. The jury then returned the verdict given above, and with that the matter may be left. History, all rights and wrongs apart, will nevertheless be interested that in a district filled with trade unionists, as this must have been, the union men should have given with such seeming unanimity their strong support to a man whose position, had it been in a trade and not in an organised profession, they would undoubtedly have stigmatised as a "blackleg."

### THE ISSUE OF FALSE DEATH CERTIFICATES.

At the Westminster Police Court last week Dr. Alexander Girvan was fined £10, and £10 costs, for describing in a certificate the death of a young domestic servant to appendicitis, peritonitis, and cardiac failure, whereas it was found that the cause of death was septic poisoning following an illegal operation; a Coroner's jury had returned a verdict of "Wilful murder" against a person or persons unknown. As a result of Dr. Spilsbury's evidence counsel for the defence said that he had advised his client that the omission of the word "miscarriage" from the death certificate had placed him legally in a false position. His motive was the girl's urgent request to conceal her condition; his treatment had been the best he knew how to give. He submitted that subsequent circumstances alone had revealed how important an omission might become. For eighteen years he had pursued his profession honestly. Mr. Horace Smith said that the offence was a grave one, with the motive for which he had nothing to do. The desirability of truth in such declarations is so obvious that this case can hardly enforce it, but it is in the instances where no tragic circumstances are involved, in the ordinary daily granting of such certificates as a matter of professional routine, without adequate personal responsibility on the part of the medical man who signs them, that stringency is needed. The average death certificate is as scientifically loose as it may well be, and far too often granted in the absence of personal inspection of the deceased.

### INSURANCE ACT AND INFIRMARY PATIENTS.

Dr. Albert Salter has drawn attention to the serious effect of the Insurance Act upon Poor-Law infirmaries. It was generally thought that the Act would reduce the number of patients in infirmaries, but the figures are about the same at the present time as they were twelve months ago. Dr. Salter, who is a member of the Bermondsey Board of Guardians, told his colleagues that the National Insurance Act had resulted in a steady stream of tuberculosis patients becoming paupers. These cases were found out at a much earlier stage than formerly. They had to wait seven weeks on the average before they were admitted to a sanatorium, where they were only retained by the London Insurance Commissioners two months. Many came back partly cured, branded as consumptives, and therefore work without, and rapidly developed the disease again. They were not eligible for further benefits under the Act, and consequently had to fall back on the only authority—the Boards of Guardians—who made provision for them. Next year he anticipated a much larger number of insured persons coming into the infirmaries. Some attempt must be made to provide benefits for these patients so long as they can be properly required. Indeed, if this be not done, the Poor Law will be compelled to fill the gap efficiently, as it is unofficially filling it to-day.

### DOCTOR AND BURSAR IN ONE.

In a sense the medical officers to public schools are on a footing which almost makes them institutional doctors, especially where a resident whole-time medical officer is appointed. They have, for instance, the charge of some kind of isolation building (frequently called a sanatorium) for the reception of infectious cases. They have distinctly public health duties to perform, they have regular hours of work, fixed salaries, and many other of the functions which distinguish institutional from private practice. An advertisement of such a vacancy in a certain Scottish school has appeared several times just recently in the daily press, with the unusual proviso added that the selected candidate must be prepared to act as bursar as well as medical officer. The business capacity of the natives of Scotland is well known to be much superior to that of Englishmen; so possibly Scottish medical men are better qualified to act as bursars than English ones are. Certainly on this side of the Border not one doctor in fifty could safely be entrusted with duties requiring any knowledge of business, nor would any school governing board think of allowing the medical officer to exercise bursarial functions. But doubtless the authorities of Trinity College, Glenalmond, know what they are about, and we trust their faith will be justified by the result.

### THE ORPHANAGE IN INSTITUTIONAL LIFE.

Orphanages and children's homes of various types which voluntary charity has created, though an exceedingly numerous class of institution, have not levelled up to nearly the same extent, and consequently have not received the same public recognition as other sections of the institutional world. A change for the better, however, seems to be taking place, so far at least as London institutions are concerned, through the agency of the Social Welfare Association for London, which formed last spring a committee of inquiry, under the chairmanship of Lord Lichfield, to investigate the work of the orphanages, children's homes, and allied institutions in the Metropolis. A detailed report on the voluntary orphanages and homes of London is now practically ready, in which, we understand, the committee's conclusions as to the needs of London's children, the extent to which voluntary charity, local authorities, and central authorities meet them; the question of greater co-operation between existing agencies; and the lines of further legislation will be set forth. An inquiry office of child welfare, where all persons requiring advice as to the agencies which exist to meet particular cases, it is hoped to establish; and this, if successful, will exist to answer questions relating to children in the same way as The Hospital's *Bureau of Information* was established to supply expert advice on matters affecting the whole institutional world. We confess that such work as the Association is doing is likely to be far more solidly useful, though less publicly advertised, than the inception of Health Week schemes and other fancies that rise—and disappear—like froth on the top of the healthy activities of the institutional world and public health. The beginning of modern hospital advance was the co-ordination of the separate institutions and the formation of such bonds of union as the great Funds, the Uniform System of Accounts, and the hospitals associations. The orphanages and child homes should also come into line, adopt the Uniform System invariably—and to secure that the influence of great Funds is invaluable—and take their share in institutional life by vieing with the infirmary, the voluntary hospital, the mental hospital, and the sanatorium in exchanging their views, difficulties, and appointments through the medium of The Hospital, which has already in its Special Numbers given them as much publicity as they were educated enough to appreciate. But orphanages have never yet realised that appeals are not the only things that should be published.

### THE PROPOSED AMALGAMATION OF ST. GEORGE'S AND WESTMINSTER HOSPITALS.

The amalgamation of these two hospitals is now a matter of grave doubt owing to a serious difference of opinion which has arisen on the question of site. St. George's Hospital is in favour of the Wandsworth site, while the Westminster Hospital favours that at Clapham.

### ST. GEORGE'S HOSPITAL.

In the *Times* of December 9 a notice appears of a special court of governors to be held at the hospital on the 19th inst., at 3 p.m., "to consider proposals made by the House Committee for the management of the hospital, which involve additional expenditure, and to sanction the purchase of the site at Wandsworth Bridge." Mr. West, the present treasurer, has resigned, and the special meeting has been called under Law 23 on the requisition of certain governors, who are of opinion that the proposal of the House Committee to retire the present secretary on his present salary, and to appoint Mr. West secretary and superintendent at a salary of £600 a year, with an additional £150 a year in lieu of board and service, with the use of a house near the hospital, the rent of which has been, we understand, some £400 a year, is inadvisable and contrary to the best interests of St. George's Hospital. The increasing losses on income, and the facts, as a correspondent pointed out last week, that in the last decade the expenditure has exceeded the income by over £50,000, although in 1907 more than £63,000 was received in legacies, coupled with the knowledge that St. George's Hospital urgently needs about £16,000 more revenue to pay its way, should have made the House Committee pause before agreeing to impose an additional annual charge upon its resources of £800 a year or more. The House Committee, we understand, further proposes to appoint a medical superintendent with emoluments amounting to £440 a year, in addition to a house with board and service.

### THE GRAVE DANGER OF ST. GEORGE'S HOSPITAL.

It has become more and more apparent of late that unless the governors interfere, St. George's Hospital may be permanently crippled by differences which have been caused through the extraordinary proposals of the House Committee, which cannot add to the hospital's popularity or prevent the retirement of the many governors who are opposed to them, because they put the best interests of St. George's Hospital before all other considerations. *Truth*, in its issue of the 10th instant, properly urges that "a trained hospital secretary skilled in the collection of funds should be a *sine qua non* in a hospital which has to face a diminishing income and a recurring deficit. The needs of the institution, and not those of one of their colleagues, ought to be, in fact, the first consideration with the managers." No doubt those governors who oppose the present proposals will give strong and sufficient reason for the faith which is in them. We hope that every governor of St. George's Hospital who can possibly attend will make it his or her business to be present at the meeting at the hospital on the 19th instant at 3 p.m., so that this great institution may be saved from the serious and permanent dangers with which it is threatened by the attempt to enforce a policy, which is not good business, and that cannot be based solely on the welfare of this institution. Another reason why every governor interested in St. George's Hospital ought to attend is that, if the proposal to purchase the Wandsworth site be carried, the amalgamation with the Westminster Hospital must be abandoned.

### THE LATEST USE FOR X-RAYS.

Announcements come from Birmingham that Dr. Hall-Edwards, locally well known as a radiologist, who is also a victim to dermatitis caused by *x*-rays, has been applying a German process to the radiography of plants and insects. It seems that the use of very soft rays from very low tubes under certain conditions may produce images of such transparent objects as flowers and paper, and experiments indicate that growth of diseases in plants may thus be observed, letters written in ink (if made with a metal basis) can be read through an envelope, and the structure of insects examined without dissection. Clearly, not only botanists and detectives are likely to benefit by this latest development of radiology.

### THE CONDITIONS OF SERVICE AT LONDON MENTAL HOSPITALS.

The Mental Hospitals Committee of the London County Council is still persevering in its efforts to attract more workers to the Mental Hospital Service. At the Manor Mental Hospital the salaries of the first and second assistant medical officers have not hitherto been in accordance with the Committee's scale of salaries for the larger institutions. The salary of the first assistant has now been fixed at £385 a year, rising to £410 a year in twelve months' time, and that of the second assistant at £260 a year, rising to £300 a year by annual increments of £10. The Committee have also agreed as from next April to make the male attendants, head attendants, and inspectors at the county mental hospitals the same extra payments in respect of their having passed certain qualifying examinations which will be allowed from that date to members of the female nursing staff. These extra payments are as follows:—(*a*) On obtaining the nursing certificate of the St. John Ambulance Association £1 a year; (*b*) on passing the preliminary examination of the Medico-Psychological Association £1 a year; and (*c*) on obtaining the nursing certificate of the Medico-Psychological Association £1 a year.

### THE HOSPITAL AND INFIRMARY DOLL SHOW.

We remind our readers that the annual hospital and infirmary festival, which the *Truth* Doll Show has become, opens at the Albert Hall on Tuesday next at 4 p.m., when the Lord Mayor and the Lady Mayoress, with the Sheriffs, will attend. It will remain open on the two following days. It must always be remembered with gratitude by the hospital world that *Truth* was the first newspaper to form the idea of a toy show for hospital and infirmary children, and the first to show how to conduct such a huge project with success. Some 30,000 dolls and toys provided by the generosity of the public will be on view before finding their way to the children's wards of the hospitals and Poor-Law institutions. We trust that many institutional workers have taken some part, either by dressing dolls or subscribing to the toy fund, to ensure the success this year again of the thirty-fourth toy show. In any case, every effort should be made to attend the exhibition at the Albert Hall next week.

### A SUCCESSFUL LINEN LEAGUE.

The Preston Infirmary County Linen League, which has been in existence for two years, has now 1,350 subscribers, a striking testimony to the way in which such organisations succeed not merely in providing articles for the hospital's use, but in stimulating interest in the institution throughout the district. The success of the League now makes it possible for it to purchase not only linen, but anything for the use and comfort of the patients, as was at first hoped. We mention this as the latest instance of the value of subsidiary organisations, since all hospitals even to-day have not learnt their usefulness.

### A PHENOMENAL HOSPITAL BAZAAR.

Not long ago attention was drawn in these columns (The Hospital, October 25, 1913; p. 85) to the dire needs of the Glasgow Maternity Hospital and to the bazaar then being organised to relieve them. The great bazaar has now been held, and the takings amounted to the enormous sum of £17,000. Not only so, three magnificent benefactions came in separately, though probably the bazaar was indirectly concerned in producing them. First, two gentlemen, Messrs. A. and J. Walker, gave £2,500 in memory of their brother. Next, Mr. Yarrow presented the charity with a convalescent home on the Gareloch. And, finally, an anonymous donor gave £3,000 through Professor Munro Kerr for developing clinical and pathological research at the hospital. It must be a very long time since any bazaar for a hospital brought in such splendid sums of money; and we do not remember to have ever heard of so successful a bazaar in Scotland before.

### DOCTOR'S DEATH FROM SEPTICÆMIA.

Mr. Hubert Liston Willcox, M.R.C.S., L.R.C.P., of Warminster, has died at the early age of thirty-six from septicæmia, following on a scratched finger incurred during the performance of an operation four weeks ago. Educated at Cheltenham and King's Colleges, he first became house surgeon at King's College Hospital, and later held the post of surgeon to the local institution at Warminster. He took the conjoint diploma in 1901, and was in partnership with his father. To the hospital world there must be something tragic in the death of a doctor in the course of his work, though the lay papers have become so lavish with the terms "medical martyr" and "martyr to science" that their proper application to those engaged on some form of research involving personal risk is apt to be lost sight of. Accidents, impossible to foresee or the result of carelessness, may happen in all occupations and have originated the generic phrase "dangerous trades" to those in which they are frequent; but we do not call every shunter killed through the absence of automatic couplings a martyr, though, as this is an avoidable risk thrust on the shunter, the term could be correctly applied. But the sympathy which always goes out to those who die at their posts is the best of all tributes, and here is an instance which demands and receives it fully.

### THIS WEEK'S DRUG MARKET.

Business has again been quiet during the week, and no general improvement is expected until early in the New Year. With the approach of the operation of stock-taking, buyers are naturally not anxious to increase their stocks more than is necessary. There seems to be no reason, however, why the markets should not become active again towards the middle of next month. Opium is quiet but steady; morphine and codeine are unchanged. Cod-liver oil is dull and quotations are unaltered. The price of castor oil has an upward tendency. Menthol and Japanese oil of peppermint are practically unchanged at the recent decline. The price of saffron is well maintained at the advanced rate. Podophyllum is rather dearer. The value of ergot of rye has an upward tendency.

